# Premorbid functioning trajectories and the one-year course of cognitive performance in first-episode psychosis: a cluster analysis in PSYSCAN

**DOI:** 10.1016/j.scog.2025.100391

**Published:** 2025-09-26

**Authors:** Margot I.E. Slot, Hendrika H. van Hell, Inge Winter-van Rossum, George Gifford, Paola Dazzan, Arija Maat, Lieuwe De Haan, Benedicto Crespo-Facorro, Birte Y. Glenthøj, Colm McDonald, Thérèse van Amelsvoort, Celso Arango, Irina Falkenberg, Barnaby Nelson, Silvana Galderisi, Mark Weiser, Gabriele Sachs, Anke Maatz, Jun Soo Kwon, the PSYSCAN Consortium, Philip McGuire, René S. Kahn

**Affiliations:** a Department of Psychiatry, UMC Utrecht Brain Center, University Medical Center Utrecht, Utrecht, the Netherlands; b Department of Psychiatry, Icahn School of Medicine, Mount Sinai, New York, the United States of America; c Department of Psychiatry, University of Oxford, Warneford Hospital, Oxford, United Kingdom; d Department of Psychological Medicine, Institute of Psychiatry, Psychology & Neuroscience, King's College London, De Crespigny Park, Denmark 458 Hill, London, SE5 8AF, United Kingdom; e Amsterdam UMC, University of Amsterdam, Psychiatry, Department Early Psychosis, Amsterdam, Meibergdreef 9, the Netherlands; f Hospital Universitario Virgen del Rocio, CIBERSAM, IBiS-CSIC, Department of Psychiatry, School of Medicine, University of Sevilla, Spain; g Centre for Neuropsychiatric Schizophrenia Research (CNSR), Centre for Clinical Intervention and Neuropsychiatric Schizophrenia Research (CINS), Mental Health Centre Glostrup, Glostrup, Denmark; h University of Copenhagen, Faculty of Health and Medical Sciences, Department of Clinical Medicine, Copenhagen, Denmark; i Centre for Neuroimaging & Cognitive Genomics (NICOG), NCBES Galway Neuroscience Centre, National University of Ireland Galway, H91 TK33, Galway, Ireland; j Department of Psychiatry and Neuropsychology, Maastricht University, Maastricht, the Netherlands; k Department of Child and Adolescent Psychiatry, Institute of Psychiatry and Mental Health, Hospital General Universitario Gregorio Marañón, IiSGM, CIBERSAM, ISCIII, School of Medicine, Universidad Complutense, Madrid, Spain; l Department of Psychiatry, University of Marburg, Rudolf-Bultmann-Straße 8, D-35039, Marburg, Germany; m Orygen, Melbourne, VIC, Australia; n Centre for Youth Mental Health, University of Melbourne, Melbourne, VIC, Australia; o Department of Mental and Physical Health and Preventive Medicine, University of Campania L. Vanvitelli, Naples, Italy; p Zachai Department of Psychiatry, Sheba Medical Center, Tel Hashomer, 52621, Israel; q Tel Aviv University School of Medicine, Ramat Aviv, Israel; r Department of Psychiatry and Psychotherapy, Medical University of Vienna, 1090, Vienna, Austria; s Department of Adult Psychiatry and Psychotherapy, Psychiatric University Clinic Zurich, University of Zurich, Switzerland; t Department of Psychiatry, Hanyang University Hospital, 222-1 Wangsimni-ro, Seongdong-gu, Seoul, Republic of Korea; u Department of Neuropsychiatry, Seoul National University Hospital, 101 Daehakno, Jongno-gu, Seoul, Republic of Korea; v Institute of Human Behavioral Medicine, SNU-MRC, 101 Daehakno, Jongno-gu, Seoul, Republic of Korea; w Department of Psychosis Studies, Institute of Psychiatry, Psychology, and Neuroscience, King's College London, United Kingdom

**Keywords:** Schizophrenia, Psychosis, FEP, Cognition, Clustering, Premorbid functioning

## Abstract

**Background:**

We examined the course of cognitive performance in first-episode psychosis (FEP) compared to healthy controls (HC), and whether this varied across subgroups of patients defined by premorbid functioning (PMF) trajectories, using a clustering approach.

**Methods:**

Data were collected in 302 FEP and 136 HC subjects participating in PSYSCAN (HEALTH.2013.2.2.1-2-FEP). K-means clustering (Euclidean distance) was used to cluster longitudinal trajectories of different PMF domains simultaneously. Since PMF was assessed retrospectively using the Premorbid Adjustment Scale (PAS), findings should be interpreted with caution, although PAS ratings have shown reasonable validity against prospective data.

**Results:**

As expected, FEP showed impaired performance across all cognitive domains compared to HC. We identified four trajectories of PMF: a normal premorbid developmental trajectory (globally-normal, 21 %), stable intermediate PMF across domains (stable-intermediate, 29 %), stable poor or deteriorating PMF in the academic domain (normal-social/poor-academic, 29 %), and a globally impaired group with poor/deteriorating PMF across domains (globally-poor, 21 %). These clusters showed distinct levels of post-onset impairments in sustained visual attention, visual working memory and emotion recognition.

**Conclusions:**

This study confirms a positive association between PMF and cognitive performance in the early years following psychosis onset. It aligns with findings that individuals later diagnosed with schizophrenia already show developmental deficits/lags from childhood to early adolescence compared to normally developing children. As PMF can be considered a proxy for cognitive reserve, our results suggest that higher reserve acts as a buffer against cognitive decline and supports better performance on sustained visual attention, complex visual working memory, and aspects of emotion recognition.

## 1 Introduction

Generalized cognitive impairment can be recognized as a core feature of schizophrenia, with deficits emerging in childhood or early adolescence, before the manifestation of the first psychotic episode (Bora and Murray, 2014; Kahn, 2020; Keefe and Kahn, 2017; Kahn and Keefe, 2013; Woodberry et al., 2008). Whether and to what extent cognitive abilities further decline, stabilize or improve after psychosis onset is less clear. A meta-analysis of longitudinal studies in first episode psychosis (FEP) and healthy controls (HC) demonstrated a stable course of cognitive functioning in the early years following psychosis onset (Watson et al., 2022), but there are examples of short-term cognitive deterioration in a subgroup of patients (Amoretti et al., 2021). In addition, there is evidence to suggest continued decline after psychosis onset in specific cognitive domains, including memory, verbal knowledge and intellectual functioning (i.e., crystallized abilities), especially over the long term (Bozikas and Andreou, 2011; Zanelli et al., 2019), with patients presenting with various profiles (Carruthers et al., 2019; Lewandowski et al., 2018) and trajectories (Habtewold et al., 2020) of cognitive impairment. Even within homogeneous samples of individuals with schizophrenia, distinct cognitive subgroups have been described; around 25 % shows relatively intact cognitive function (within ~0.5 SD of the mean performance of HCs), approximately 31 % exhibits moderate or mixed cognitive impairments (within 0.5–1.5 SD below HCs), while the majority of patients (around 44 %) demonstrate severe cognitive dysfunction (>1.2 SD below HCs) (Carruthers et al., 2019).

Given the inter-individual variability in the severity of cognitive impairment after the emergence of psychosis, predictors for poor cognitive outcomes are of obvious interest. Poor premorbid functioning (PMF) has been identified as one of the most replicated predictors of poorer cognitive, clinical and functional outcomes in patients with first episode psychosis (Díaz-Caneja et al., 2015). Premorbid functioning can be defined as the level of achievement of developmental milestones expectable at various age levels, in several major areas of development, and measured before the onset of florid psychotic symptoms of schizophrenia. For the current study, premorbid functioning (PMF) is further defined as the level of functioning in the domains of sociability and withdrawal, peer relationships, scholastic performance and adaptation to school, in childhood (up to age 11) and early adolescence (ages 12–15), as measured with the Premorbid Adjustment Scale (Strauss et al., 1977). Several studies explored individual variability in PMF across developmental stages, in relation to symptomatic and functional outcomes in schizophrenia spectrum disorder, using clustering techniques. In clustering, patients are grouped based on similarity on one or more characteristics (in this context, on premorbid functioning) (Den Teuling et al., 2021). Generally, three or four clusters are found with varying levels and courses of PMF, but more clusters have been reported as well (Quee et al., 2014; Tiles-Sar et al., 2023). Patients with a normal developmental trajectory seem to respond better to treatment, and have better cognitive functioning (Rabinowitz et al., 2006), on verbal fluency and memory tasks in particular (Addington and Addington, 2005). In comparison, patients with a stable poor or deteriorating developmental trajectory display more severe positive and negative symptoms (Rabinowitz et al., 2006; Addington and Addington, 2005) and poorer social functioning (Addington and Addington, 2005) after psychosis onset. Most of the existing research explored trajectories of overall premorbid functioning, defined as the average score on the four areas of development captured by the PAS (Strauss et al., 1977; Van Mastrigt and Addington, 2002), but the need to distinguish between different components of PMF has been highlighted by a number of studies (Barajas et al., 2013; Chang et al., 2013; Ferraro et al., 2021; Monte et al., 2008; Setién-Suero et al., 2024; Tan et al., 2021). To illustrate, good premorbid *academic* functioning (scholastic performance and adaptation to school) exclusively has been associated with better learning and memory (Barajas et al., 2013). In addition, a stable course of premorbid *social* functioning (peer relationships and sociability and withdrawal) rather than premorbid academic functioning has been linked to fewer negative symptoms (Larsen et al., 2004). Moreover, the research to date has tended to focus on outcomes at a single time point rather than evaluating outcomes longitudinally (Quee et al., 2014; Larsen et al., 2004; Béchard-Evans et al., 2010; Caqueo-Urízar et al., 2022).

The present study aims to contribute to this area of research by 1) comparing the cognitive performance of patients diagnosed with first-episode psychosis in the context of a schizophrenia spectrum disorder (FEP) with that of healthy controls, 2) examining whether cognitive functioning changes over 12 months' time in these groups, and 3) investigating whether cognitive performance over time differs between FEP patients grouped on the basis of their trajectory of PMF, using an advanced clustering approach. The clustering method used provides the opportunity to study the progression of the four areas of PMF in parallel (i.e., identify “joint-variable trajectories”). Inclusion of a healthy control group not only enables a comparison with normal, age-related cognitive variation in the general population, it also allows differentiating between actual cognitive improvements and practice effects (Goldberg et al., 2010). The cognitive domains of sustained attention, visuospatial working memory, visuospatial episodic memory and associative learning, and emotion recognition were selected to capture key cognitive deficits observed in psychosis (Gifford et al., 2024; Kern et al., 2004). We hypothesize that cognitive performance may be most preserved over time in patients with good or intermediate PMF, especially for the domains of PMF that are most related to cognition, i.e. scholastic performance and adaptation to school. These domains may be reflective of or at least closely linked to the level of cognitive reserve, defined as the ability to efficiently use brain networks in order to optimize performance in response to increased cognitive demands or to cope with brain pathology (Stern, 2002).

## 2 Methods

### 2.1 Setting and participants

The present work used data from the FEP cohort (*n* = 302) and healthy control (HC) cohort (*n* = 136) from PSYSCAN (HEALTH.2013.2.2.1-2-FEP). Subjects were eligible for participation if they were 16–40 years of age and capable of providing informed consent (or assent in case of a minor). FEP patients met criteria for first-episode psychosis in the context of a schizophrenia spectrum disorder, defined by a DSM-IV diagnosis of schizophrenia, schizoaffective disorder (depressive type) or schizophreniform disorder (Diagnostic and Statistical Manual of Mental Disorders, 4th ed., text rev.) (American Psychiatric Association, 2000). Detailed eligibility criteria and recruitment and study procedures of the FEP cohort have been described previously (Slot et al., 2024; Tognin et al., 2020). The exclusion criteria for healthy controls were similar as those applied to the FEP cohort, with the addition of the following exclusion criteria: 1) Lifetime history of a DSM Axis-I psychiatric disorder, 2) At high risk of developing psychosis, based on inclusion in one of three groups as assessed by the Comprehensive Assessment of At-Risk Mental States (CAARMS version 2006) (Yung et al., 2006) and meeting criteria for “basic symptoms” as assessed using the Schizophrenia Proneness Instrument (SPI-A) (Schultze-Lutter et al., 2006), 3) First-degree relative with a lifetime history of affective or non-affective psychosis (defined by treatment or diagnosis), 4) Previous intake of antipsychotic medication, 5) Current intake of psychoactive medication, 6) IQ < 70 (see Supplementary Table 1). Written informed consent was obtained from all participants, or their legal representatives. The study was approved by the ethics committees of the participating centers and conducted in compliance with the Declaration of Helsinki (2013) and the International Committee on the Harmonization of Good Clinical Practice, if applicable according to local laws and regulations. Subjects were recruited at institutions situated in Europe, Israel, Australia and South-Korea. The study was monitored by the University Medical Center Utrecht, the Netherlands. The last study visit took place on August 12, 2020, as some final visits were delayed due to COVID-19 lockdown restrictions.

### 2.2 Study design

Both cohorts were followed for one year in a naturalistic design. Premorbid functioning was estimated at baseline using the (short version of the) Premorbid Adjustment Scale (PAS, short version validated by Van Mastrigt & Addington) (Van Mastrigt and Addington, 2002; Cannon-Spoor et al., 1982). The short PAS is a rating scale designed to retrospectively measure premorbid functioning in childhood (up to 11 years), early adolescence (12–15 years), late adolescence (16–18 years) and adulthood (19+ years), based on a semi-structured clinical interview. The PAS has been widely used and has demonstrated acceptable predictive and concurrent validity (Brill et al., 2008). The PAS differentiates between five domains of functioning which are rated up to one year before illness onset: sociability and withdrawal, peer relationships, scholastic performance, adaptation to school, and social-sexual aspects of life. The social-sexual domain is rated for the adolescence and adulthood periods only. In PSYSCAN, only the childhood and early adolescent period were used, to reduce the risk of evaluating early morbidity instead of premorbid functioning. A semi-structured clinical interview was used to score the PAS domains on a 0–6 rating scale, with higher scores indicating poorer premorbid functioning. An overview of sample questions from the interview is provided in Supplementary Table 2. For all clusters, the median PAS and change scores between childhood and early adolescence were labelled in accordance with previously proposed criteria (Larsen et al., 2004); a median PAS score below 1.5 is considered good, a score equal to or higher than 1.5 but lower than 3.0 is considered intermediate, and a score of 3 and higher is considered poor. The trajectories of the clusters were defined as stable between childhood and early adolescence (change score < 1), slightly deteriorating (change score ≥ 1 and < 2) and clearly deteriorating (change score ≥ 2).

Cognition was assessed at baseline and at six and twelve months after study entry. A computerized battery of neuropsychological tests was used, ensuring consistency in test administration and minimizing rater variance. All tasks were performed on an iPad using a battery of tests developed by Cambridge Cognition (https://www.cambridgecognition.com/cantab), capturing key deficits associated with psychosis. Tests were administered in the following order: 1) the Emotion Recognition Task (ERT) was used to measure the participant's ability to identify the basic emotions sadness, happiness, fear, anger, disgust and surprise in facial expressions; 2) the Paired Associate Learning Task (PAL) was used to assess visuospatial episodic memory and associative learning; 3) the Spatial Span Task (SSP) assessed visuospatial working memory; and 4) the Rapid Visual Information Processing task (RVP) was used to measure sustained visual attention. A detailed description of the cognitive subtests is provided in the Supplementary Methods. Total administration time was approximately 25 min.

In addition to the computerized cognitive test battery, an IQ assessment was conducted at baseline using an abbreviated version of the Wechsler Adult Intelligence Scale-III (WAIS-III) (Wechsler, 1997; Blyler et al., 2000). Four subtests were performed: Digit Symbol Substitution (psychomotor speed, perception, working memory, sustained attention, visuomotor coordination), Arithmetic (attention and working memory), Block Design (visuospatial abilities, executive functioning, visuomotor coordination, perception) and Information (crystallized knowledge, retrieval from long-term memory).

### 2.3 Statistical analysis

Data were analyzed using R Statistical Software version 4.4.0 (R Core Team, 2024). Cognitive assessments with missing tests due to the COVID-19 pandemic were excluded from analysis (FEP = 16, HC = 10). Data were visually inspected to check for outliers and violation of assumptions. Extreme values were excluded as appropriate (scores out of the possible value ranges for a given task). RVP, ERT and SSP data were removed from the analysis if 0 attempts were made during the task, and PAL data were removed if a participant reached less than 2 patterns. The PAS domain social-sexual aspects of life was excluded from the analyses to meet criteria for longitudinal clustering and facilitate comparison between premorbid functioning in childhood and early adolescence. For each premorbid developmental period, a total score was determined by dividing the sum of the four remaining domains by the highest possible score for the items rated. The overall PAS score was calculated by averaging the childhood and early adolescence PAS scores. Cluster analyses were performed using the package KmL3D version 2.4.6.1 (Genolini et al., 2023, Genolini et al., 2015), a version of k-means clustering designed to cluster longitudinal trajectories of different variables simultaneously (Genolini et al., 2013), using the Euclidean distance. It summarizes the two correlated scores (childhood score and early adolescence score) on the four PAS domains (the joint-trajectories) into a single nominal variable (a group/cluster), which captures the information from the underlying correlated scores (Genolini et al., 2013), thereby allowing the exploration of relationships between the course of different components of PMF. Following previous literature, two-, three- and four-cluster solutions were explored by running the (non-parametric) k-means clustering algorithm 20 times for each number of clusters. To determine the optimum number of clusters, quality criteria for non-parametric data were compared (three variants of the Calinski and Harabasz) (Calinski and Harabasz, 1974; Genolini et al., 2015; Kryszczuk and Hurley, 2010). Following the subdivision in previous studies (Chang et al., 2013; Ferraro et al., 2021; Ayesa-Arriola et al., 2013; Rund et al., 2004), in the interpretation of the results, the four PAS domains were further categorized into a social (peer relationships and sociability and withdrawal) and an academic component (scholastic performance and adaptation to school).

In line with the methodology used by Gifford et al. (2024), raw cognitive test scores of the FEP and HC cohort were converted into z-scores based on an external healthy control sample matched in age and sex, provided by Cambridge Cognition. Normative z-scores were not available for participants below the age of 18 years. Linear mixed models were used to examine longitudinal changes in cognitive performance between groups. To control for the potential effect of the group difference in years of education on cognitive performance, years of education was included as a covariate in the FEP versus HC group comparisons. To control for sex differences in premorbid functioning and cognitive performance, sex was included as a covariate in the cluster comparisons (Leung and Chue, 2000; Ventura et al., 2023a). The inclusion of years of education and sex as covariates was based on prior literature showing effects of education and sex on premorbid functioning and cognitive performance (Weiser et al., 2000; Ventura et al., 2023b; Swanson et al., 1998; Cámara et al., 2021; Ayesa-Arriola et al., 2018; Allen et al., 2013). The standardized scores are already adjusted for both age and sex, ensuring that age-related variance was accounted for at the stage of score transformation. All tests were two-tailed and the significance level was set to α = 0.05. Post-hoc tests were adjusted for multiple comparisons (either Tukey or Bonferroni correction). Exploratory analyses of specific emotions on the ERT were based on raw scores instead of z-scores, as standardized scores were not available.

## 3 Results

302 patients (67.2 % male) and 136 HC participants (59.6 % male) were included. Patients had a mean age of 25.3 (SD = 5.7) and an average of 14.2 educational years (SD = 3.1). Healthy controls were younger (mean = 23.8, SD = 4.3, *p* = .002) and had more years of education (mean = 16.0, SD = 3.0, *p* < .001). IQ was significantly lower in FEP participants (mean = 90.8, SD = 18.6) compared to healthy controls (mean = 112.2, SD = 16.0), *p* < .001. The FEP cohort had a median illness duration of 9.4 months (defined as the time between the date of first acceptance at healthcare service for psychosis and study entry, IQR = 9.3), and a mean PANSS total score of 55.2 (SD = 16.8) at baseline. Detailed sociodemographics and clinical characteristics of the two cohorts are included in Supplementary Table 4. A comprehensive clinical description of the FEP cohort has been provided elsewhere (Slot et al., 2024).

**Fig. 1:**
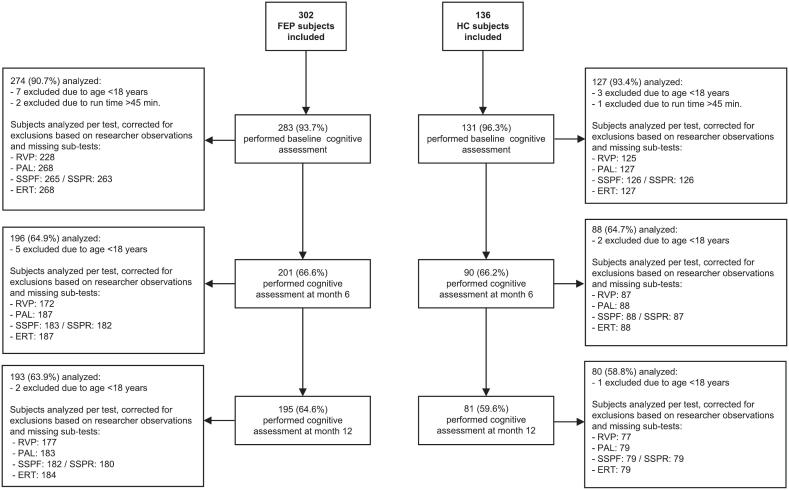
Flowchart of participants in the cognitive assessments.

Of the 302 FEP participants, 214 (70.9 %) completed the 6-month and 226 (74.8 %) the 12-month follow-up visits. A total of 97 (71.3 %) and 91 (66.9 %) of the 136 HC subjects completed the 6- and 12-month follow-up visits, respectively. An overview of the number of cognitive assessments per visit is provided in Fig. 1. Several test scores were excluded due to researcher observations (e.g. signs of non-adherence or non-comprehension of instructions, see Supplementary Table 3).

### 3.1 Cognitive performance in patients versus healthy controls

In line with previously reported findings on PSYSCAN data (Gifford et al., 2024), linear mixed models corrected for years of education revealed lower cognitive performance in FEP than healthy controls on all cognitive domains (ranging from *p* < .0001 to *p* = .0003, with η^2^p ranging from 0.01 to 0.17). The forward spatial span and emotion recognition were stable over 12 months' time in both groups (*p* = .2519, η^2^p = 0.0049, and *p* = .1437, η^2^p = 0.0060, respectively). In contrast, the reverse spatial span (SSP) and performance in the domains of sustained visual attention (RVP) and visuospatial episodic memory and associative learning (PAL) improved over 12 months in both groups (ranging from *p* < .0001 to *p =* .0072, with η^2^p ranging from 0.02 to 0.19). Detailed results are included in Supplementary Table 5 and Supplementary Fig. 1.

### 3.2 Clusters of premorbid functioning

**Fig. 2:**
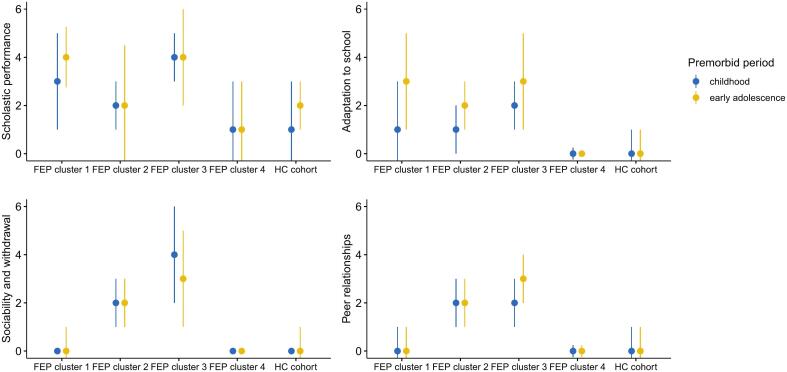
Grouped error plots showing the distribution of scores on the different domains of premorbid functioning (median and interquartile range) across the groups, separated per premorbid developmental period. Higher scores indicate worse premorbid functioning.

PAS scores were available for 293 patients (97.0 %) and 128 healthy controls (94.1 %). Five patients were removed from the clustering analysis due to missing data on PAS subitems. The non-parametric quality criteria favored a cluster solution of four premorbid functioning trajectories (i.e., the Genolini (Genolini et al., 2015) and the Kryszczuk variant (Kryszczuk and Hurley, 2010) of the Calinski & Harabasz criterion (Calinski and Harabasz, 1974) were maximized and the Davies & Bouldin criterion (Davies and Bouldin, 1979) was minimized). The joint trajectories on the different PAS items are displayed in Fig. 2. Cluster 1 (normal-social/poor-academic, 29.2 % of the FEP sample) was defined by a normal developmental trajectory in the social domains (i.e., sociability and withdrawal and peer relationships), but a consistently poor or deteriorating trajectory in academic domains (i.e., scholastic performance and adaptation to school). Cluster 2 (stable-intermediate, 28.8 %) was distinguished by intermediate but stable premorbid functioning across all domains. Cluster 3 (globally-poor, 21.2 %) was characterized by stable poor or deteriorating premorbid functioning across all domains (i.e., the changes from childhood to early adolescence implied by the figure (in sociability and withdrawal and peer relationships) are not statistically significant, *p* > .05). Cluster 4 (globally-normal, 20.8 %) was characterized by a normal premorbid developmental trajectory, similar to that in the healthy control group.

Baseline characteristics of the different clusters as well as the healthy control cohort are presented in Table 1. The majority of the sample in general was male (67.0 %), but males were even more overrepresented in cluster 1 (81.0 %, *p* = .001). Approximately 60 % of the patients in clusters 2 and 4 were either employed or pursuing education, compared to 40 % in clusters 1 and 3. The severity of psychotic symptoms was higher, and social-occupational functioning was generally lower in patients from cluster 3 than in the remaining clusters at baseline (*p* < .001). Patients from clusters 1 and 3 had a lower estimated IQ (mean ranging from 84.6 to 85.7, SD ranging from 16.5 to 18.4) than clusters 2 and 4 (mean = 96.0–98.1, SD = 15.8–19.7).

**Table 1:** Baseline characteristics of FEP patients clustered on longitudinal trajectories of premorbid functioning versus healthy controls.

	FEP cluster 1 (normal-social/poor-academic)	FEP cluster 2 (stable-intermediate)	FEP cluster 3 (globally-poor)	FEP cluster 4 (globally-normal)	HC cohort	Test statistic	*p*-Value	N	Post-hoc tests *
	*n = 84*	*n = 83*	*n = 61*	*n = 60*	*n = 135*				
Sex						χ^2^ = 15.78	0.003	423	*a, d*
Female	16 (19.0 %)	38 (45.8 %)	22 (36.1 %)	19 (31.7 %)	55 (40.7 %)				
Male	68 (81.0 %)	45 (54.2 %)	39 (63.9 %)	41 (68.3 %)	80 (59.3 %)				
Age (years)	25.0 (5.2)	26.1 (6.0)	24.8 (5.6)	24.8 (5.5)	23.8 (4.3)	*F* = 2.63	0.034	422	*g*
Years of education **	13.5 (3.1)	14.8 (2.9)	13.1 (2.7)	15.1 (3.0)	16.0 (3.0)	*F* = 14.47	<0.001	422	*a, c, d, e, h, i*
Educational level ***							.	421	
Less than high school	8 (9.5 %)	2 (2.4 %)	5 (8.2 %)	0 (0.0 %)	0 (0.0 %)				
High school	38 (45.2 %)	29 (34.9 %)	24 (39.3 %)	20 (33.3 %)	14 (10.5 %)				
Professional training	23 (27.4 %)	22 (26.5 %)	19 (31.1 %)	10 (16.7 %)	24 (18.0 %)				
University	14 (16.7 %)	28 (33.7 %)	12 (19.7 %)	27 (45.0 %)	76 (57.1 %)				
Post-graduate university	1 (1.2 %)	2 (2.4 %)	1 (1.6 %)	3 (5.0 %)	19 (14.3 %)				
Employment						χ^2^ = 77.77	<0.001	400	*d, g, i, j*
Employed or student	35 (41.7 %)	47 (56.6 %)	22 (36.1 %)	37 (61.7 %)	104 (92.9 %)				
Unemployed	49 (58.3 %)	36 (43.4 %)	39 (63.9 %)	23 (38.3 %)	8 (7.1 %)				
Time since treatment initiation for psychosis (months), *Mdn (IQR)*^†^	4.9 (10.6)	6.6 (16.4)	6.6 (13.9)	4.7 (11.9)		*H* = 1.24	0.744	288	
DUP (months), *Mdn (IQR)*^‡^	0.9 (2.6)	1.0 (5.8)	1.3 (4.8)	0.9 (4.7)		*H* = 1.64	0.650	288	
PAS total, *Mdn (IQR)*	1.6 (0.7)	1.9 (0.6)	3.0 (0.8)	0.5 (0.4)	0.6 (0.8)	*H* = 301.6	<0.001	415	
PANSS total	52.2 (15.1)	54.1 (16.0)	64.0 (18.1)	50.1 (16.1)	31.7 (2.2)	*F* = 73.48	<0.001	411	*b, d, e, g, h, i, j*
PANSS positive	12.0 (4.8)	12.8 (5.5)	15.1 (5.8)	12.1 (5.8)	7.1 (0.4)	*F* = 41.79	<0.001	414	*b, d, e, g, h, i, j*
PANSS negative	13.6 (5.8)	14.0 (6.0)	17.6 (7.6)	12.5 (5.2)	7.3 (0.7)	*F* = 65.32	<0.001	413	*b, d, e, g, h, i, j*
PANSS general	26.6 (7.5)	27.3 (8.0)	31.3 (9.3)	25.6 (7.9)	17.3 (1.8)	*F* = 56.32	<0.001	412	*b, d, e, g, h, i, j*
CGI-S, *Mdn (IQR)*	3.0 (2.0)	4.0 (1.0)	4.0 (2.0)	3.0 (2.0)	1.0 (0.0)	*H* = 224.37	<0.001	399	*b, d, g, i, j*
HDRS score, *Mdn (IQR)*	5.0 (7.0)	6.0 (6.0)	7.0 (10.0)	4.0 (4.5)	1.0 (2.0)	*H* = 129.33	<0.001	412	*d, g, i, j*
GAF score	58.3 (17.1)	54.0 (19.9)	46.1 (16.6)	58.7 (20.9)	85.1 (7.2)	*F* = 89.09	<0.001	417	*b, d, e, g, h, i, j*
SOFAS score	55.7 (16.6)	54.7 (19.1)	46.9 (14.2)	60.7 (20.1)	85.2 (6.5)	*F* = 99.92	<0.001	417	*b, d, e, g, h, i, j*
WAIS total IQ	85.7 (18.4)	96.0 (19.7)	84.6 (16.5)	98.1 (15.8)	112.2 (16.0)	*F* = 41.54	<0.001	411	*a, c, d, e, g, h, i, j*
WAIS arithmetic	7.7 (3.5)	8.9 (3.6)	7.2 (3.7)	10.1 (3.5)	11.3 (3.8)	*F* = 19.64	<0.001	412	*c, d, g, h, i*
WAIS symbol substitution	7.0 (2.8)	8.4 (3.2)	6.7 (2.8)	8.6 (3.0)	11.2 (3.1)	*F* = 35.90	<0.001	412	*a, c, d, e, g, h, i, j*
WAIS information	9.2 (3.9)	10.7 (3.7)	9.1 (3.8)	11.2 (3.6)	12.0 (3.3)	*F* = 10.76	<0.001	411	*c, d, h, i*
WAIS block design	7.8 (3.6)	9.9 (4.4)	8.3 (3.6)	8.8 (3.7)	12.3 (3.0)	*F* = 26.32	<0.001	412	*a, d, g, i, j*

Note. Data are n (%) or mean (SD), unless otherwise indicated. P-value of ANOVA (continuous variable normal distributed), Kruskal-Wallis test (continuous variable non-normal distributed) or Chi-Square test (categorical variable). *a* Significant difference between Cluster 1 and 2. *b* Significant difference between Cluster 1 and 3. *c* Significant difference between Cluster 1 and 4. *d* Significant difference between Cluster 1 and HC. *e* Significant difference between Cluster 2 and 3. *f* Significant difference between Cluster 2 and 4. *g* Significant difference between Cluster 2 and HC. *h* Significant difference between Cluster 3 and 4. *i* Significant difference between Cluster 3 and HC. *j* Significant difference between Cluster 4 and HC. * *P*-values post-hoc tests adjusted for multiple comparisons (Bonferroni). ** Years of education = years in school and college/university (not including kindergarten/nursery). *** Each category reflects individuals who finished as well as individuals who did not (yet) finish this level of education. ^†^ Treatment initiation is defined as the date of first acceptance at healthcare service for psychosis (this could be either inpatient or outpatient, depending on the setting to which the individual first presented for psychosis). ^‡^ Duration of untreated psychosis (DUP) is defined as the time interval between first onset of frank psychotic symptoms and the date of first acceptance at healthcare service for psychosis. FEP = First Episode Psychosis in the context of a Schizophrenia-Spectrum Disorder. PAS = Premorbid Adjustment Scale. PANSS = Positive and Negative Syndrome Scale (score range: positive (7–49), negative (7–49), general (16–112), total (30−210); higher scores indicate more severe psychotic symptoms). CGI-S = Clinical Global Impression – Severity (1–7, a higher score indicates more severe illness). HDRS = Hamilton Depression Rating Scale (0–50, higher scores indicate more severe depressive symptoms). GAF = Global Assessment of Functioning (1–100, higher scores indicate a higher level of functioning). SOFAS = Social and Occupational Functioning Assessment Scale (1–100, higher scores indicate a higher level of functioning). WAIS = Wechsler's Adult Intelligence Scale, Third Edition, Short Form.

### 3.3 Rapid Visual Information Processing task

**Fig. 3:**
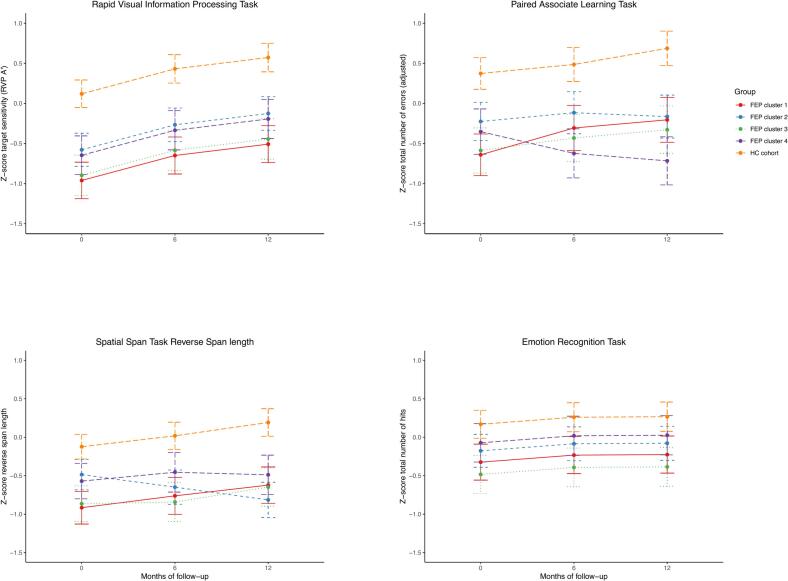
Cognitive performance over a one-year period in FEP participants clustered on premorbid functioning in childhood and early adolescence, compared to healthy controls (HC). Results of repeated measures mixed model analyses, corrected for sex.

Linear Mixed Models corrected for sex revealed a significant effect of time (*F*(2,486.3) = 56.24, *p* < .0001, η^2^p = 0.19) and group (*F*(4,379.3) = 29.08, *p* < .0001, η^2^p = 0.23) on sustained visual attention (Table 2 and Fig. 3). Cognitive performance on the RVP improved over 6 and 12 months in all groups (*p* < .0001). However, each FEP cluster performed consistently worse than healthy controls (*p* < .0001). The lowest performance was observed in cluster 1 (normal-social/poor-academic), with scores differing significantly from cluster 2 (stable-intermediate), *p* = .0349.

**Table 2:** Linear mixed model results of longitudinal changes in cognitive performance in FEP participants clustered on premorbid functioning in childhood and early adolescence, as well as healthy controls (HC). Analyses are corrected for sex.

Outcome variable	Primary analyses					Pairwise comparisons			
	*F*-statistic		Df	*p*-Value	Partial *η*^*2*^		*t*-Statistic	Df	*p*-Value
RVP	Time point:	56.24	2, 486.3	<0.0001	0.19	Month 0 < Month 6	−7.1	488	<0.0001
						Month 0 < Month 12	−10.2	502	<0.0001
						Month 6 < Month 12	−3.1	470	0.0060
	Group:	29.08	4, 379.3	<0.0001	0.23	Cluster 1 < Cluster 2	−2.9	394	0.0349
						Cluster 1 < HC	−9.2	387	<0.0001
						Cluster 2 < HC	−6.0	388	<0.0001
						Cluster 3 < HC	−7.6	371	<0.0001
						Cluster 4 < HC	−6.0	383	<0.0001
	Sex:	0.18	1, 376.9	0.669	0.005				
PAL	Time point * group:	4.03	8, 527.2	0.0001	0.06	Cluster 1 (M00) < HC cohort (M00)	−7.2	586	<0.0001
						Cluster 1 (M06) < HC cohort (M06)	−5.0	740	0.0001
						Cluster 1 (M12) < HC cohort (M12)	−5.6	732	<0.0001
						Cluster 2 (M00) < HC cohort (M00)	−4.2	596	0.0024
						Cluster 2 (M06) < HC cohort (M06)	−3.8	740	0.0116
						Cluster 2 (M12) < HC cohort (M12)	−5.3	762	<0.0001
						Cluster 3 (M00) < HC cohort (M00)	−6.1	587	<0.0001
						Cluster 3 (M06) < HC cohort (M06)	−5.4	680	<0.0001
						Cluster 3 (M12) < HC cohort (M12)	−6.0	688	<0.0001
						Cluster 4 (M00) < HC cohort (M00)	−4.7	590	0.0004
						Cluster 4 (M06) < HC cohort (M06)	−6.4	728	<0.0001
						Cluster 4 (M12) < HC cohort (M12)	−8.3	709	<0.0001
						Cluster 1 (M00) < Cluster 1 (M12)	−3.9	564	0.0083
						HC cohort (M00) < HC cohort (M12)	−3.6	555	0.0230
	Sex:	5.23	1, 382.4	0.0227	0.01				
SSP forward	Time point:	1.13	2, 541.3	0.3252	0.004	Cluster 1 < HC cohort	−7.5	391	<0.0001
	Group:	25.63	4, 367.9	<0.0001	0.22	Cluster 2 < HC cohort	−6.5	400	<0.0001
						Cluster 3 < HC cohort	−7.8	372	<0.0001
						Cluster 4 < HC cohort	−6.3	383	<0.0001
	Sex:	3.50	1, 366.3	0.0620	0.01				
SSP backward	Time point * group:	3.04	8, 535.5	0.0024	0.04	Cluster 1 (M00) < HC cohort (M00)	−6.7	673	<0.0001
						Cluster 1 (M06) < HC cohort (M06)	−5.7	810	<0.0001
						Cluster 1 (M12) < HC cohort (M12)	−6.0	800	<0.0001
						Cluster 2 (M06) < HC cohort (M06)	−6.2	811	<0.0001
						Cluster 2 (M12) < HC cohort (M12)	−7.3	820	<0.0001
						Cluster 3 (M00) < HC cohort (M00)	−5.7	675	<0.0001
						Cluster 3 (M06) < HC cohort (M06)	−5.9	778	<0.0001
						Cluster 3 (M12) < HC cohort (M12)	−5.8	772	<0.0001
						Cluster 4 (M00) < HC cohort (M00)	−3.5	683	0.0404
						Cluster 4 (M06) < HC cohort (M06)	−3.2	802	(0.0855)
						Cluster 4 (M12) < HC cohort (M12)	−4.6	800	0.0004
						HC cohort (M00) < HC cohort (M12)	−3.7	555	0.0156
	Sex:	2.54	1, 376.3	0.1115	0.0089	Cluster 1 (M00) < Cluster 2 (M00)	−3.2	686	(0.0895)
ERT	Time point:	1.89	2, 555.8	0.1525	0.0056	Cluster 1 < HC cohort	−4.1	395	0.0005
	Group:	7.93	4, 382.1	<0.0001	0.08	Cluster 2 < HC cohort	−2.9	402	0.0356
						Cluster 3 < HC cohort	−4.9	375	<0.0001
						Cluster 3 < Cluster 4	−2.6	374	(0.0652)
	Sex:	2.11	1, 381.3	0.1468	0.0043				

Note. Pairwise contrasts were corrected for multiple comparisons using the Tukey method. Only significant pairwise comparisons are shown, with p-values for trends indicated in brackets (). RVP = Rapid Visual Information Processing Task. PAL = Paired Associate Learning Task. SSP = Spatial Span. ERT = Emotion Recognition Task.

### 3.4 Paired Associate Learning Task

A significant time*group interaction was observed on the total number of errors on the PAL, *F*(8,527.2) = 4.03, *p* = .0001, η^2^p = 0.06 (Table 2 and Fig. 3). The four clusters performed worse than healthy controls at each time point (ranging between *p* < .0001 and *p* = .0116). Cognitive performance on the PAL remained stable over time in all groups, except cluster 1 (normal-social/poor-academic) and healthy controls, who showed a slight improvement in visuospatial episodic memory and associative learning over 12 months' time (*p* = .0083 and *p* = .0230 respectively).

### 3.5 Spatial Span Task

Linear mixed model analyses demonstrated a significant main effect of group on the forward spatial span length (*F*(4,367.9) = 25.63, *p* < .0001), η^2^p = 0.22), but performance was stable over time (*F*(2,541.3) = 1.13, *p* = .3252). The forward spatial span was lower in all FEP clusters compared to healthy controls (*p* < .0001, Table 2). Linear mixed models of the reverse spatial span length revealed a significant time*group interaction (*F*(8,535.5) = 3.04, *p* = .0024, η^2^p = 0.04, Table 2 and Fig. 3). Clusters 1 (normal-social/poor-academic) and 3 (globally-poor) had a lower reverse spatial span than healthy controls at each time point (*p* < .0001). Cluster 2 (stable-intermediate) performance was lower than healthy controls at month 6 and 12 only (*p* < .0001), and cluster 4 (globally-normal) only differed from healthy controls at month 0 (*p* = .0404) and month 12 (*p* = .0004). The reverse spatial span increased over 12 months' time in healthy controls exclusively (*p =* .0230).

### 3.6 Emotion Recognition Task

Throughout the follow-up period, the total number of hits on the ERT remained stable in all groups (*F*(2,555.8) = 1.89, *p* = .1525). Linear mixed models revealed a significant group effect (*F*(4,382.1) = 7.93, *p* < .0001, η^2^p = 0.08, Table 2 and Fig. 3). Clusters 1 (normal-social/poor-academic), 2 (stable-intermediate) and 3 (globally-poor) performed significantly worse on the emotion recognition task than healthy controls (ranging between *p* < .0001 and *p* = .0356). Individuals in cluster 4 (globally-normal) performed similar to healthy controls (*p* = .3584). Exploratory linear mixed models on the different types of emotion indicated that the number of hits (raw scores) for the recognition of sadness and surprise was worse in clusters 1 (normal-social/poor-academic), 2 (stable-intermediate) and 3 (globally-poor) compared to healthy controls (Supplementary Table 6 and Supplementary Fig. 2). Emotion recognition was found to be most impaired in cluster 3 (globally-poor), which additionally showed difficulties in recognizing happiness, anger and disgust compared to controls.

## 4 Discussion

The current study explored the early course of cognitive performance in relation to premorbid functioning in a naturalistic sample of patients diagnosed with first-episode psychosis. In general, patients showed impaired performance across all cognitive domains compared to healthy controls. Over a 12-month period, small improvements were observed in the domains of visuospatial working memory, visuospatial episodic memory and associative learning, and sustained visual attention in both groups, likely reflecting practice effects (Watson et al., 2022). A clustering approach revealed four trajectories of premorbid functioning in FEP, which were associated with different levels of post-onset cognitive impairment across several domains. Our results corroborate previous findings that individuals later diagnosed with schizophrenia already show developmental deficits or lags from childhood to early adolescence compared to normally developing children (Mollon and Reichenberg, 2018; Reichenberg et al., 2010). It also aligns with literature reporting a positive association between the level of premorbid functioning and cognitive outcomes following psychosis onset (Addington and Addington, 2005; Setién-Suero et al., 2024; Tan et al., 2021; Larsen et al., 2004; Bucci et al., 2018; Stefanatou et al., 2018) and suggests that heterogeneity in cognitive outcomes in FEP may be related to the level and course of functioning in the period prior to the onset and prodromal phase of illness. As premorbid functioning is considered a proxy measure of cognitive reserve (Amoretti et al., 2022; Buonocore et al., 2019; Herrero et al., 2020; Rodriguez et al., 2022), this may indicate that a higher level of cognitive reserve could explain better cognitive performance in FEP.

### 4.1 Course of premorbid functioning

Cluster 1 (normal-social/poor-academic) was characterized by a normal social developmental trajectory but a consistently poor or deteriorating trajectory of academic PMF. The lowest level of PMF was observed in cluster 3 (globally-poor), with deteriorating or poor functioning in all domains of premorbid development. Clusters 1 and 3 had a 10-point lower estimated IQ at baseline compared to clusters 2 and 4. Cluster 3 was distinguished from cluster 1 by more severe psychotic symptoms and lower social-occupational functioning after psychosis onset (in addition to the differences in PMF). Cluster 2 (stable-intermediate) was characterized by a stable course of intermediate PMF in all domains. Cluster 4 (globally-normal) showed a normal premorbid developmental trajectory, almost identical to the healthy control group, although we cannot preclude the possibility that this subgroup also experienced a decline in premorbid functioning, which only became apparent in late adolescence. Interestingly, clusters with a higher level of premorbid functioning (cluster 2 and 4) scored better on other measures that are closely linked to cognitive reserve as well (Buonocore et al., 2019; Amoretti et al., 2016; De la Serna et al., 2023; Sánchez-Torres et al., 2023); they had received more years of education, were more often employed or pursuing education (i.e., 60 % in clusters 2 and 4 compared to 40 % in clusters 1 and 3), and demonstrated better performance on the WAIS information subtest, which can be used to estimate premorbid IQ (Rodriguez et al., 2022; Elkana et al., 2020).

### 4.2 Cognitive functioning across clusters

The level of cognitive impairment slightly differed between clusters; more severe deficits in sustained visual attention (RVP) and visual working memory (reverse SSP) seem to be related to poorer academic premorbid functioning. The reverse spatial span task invokes complex cognitive processes such as the manipulation of information stored in working memory (Harvey, 2019). The rapid visual processing task also requires additional cognitive processes beyond attention, such as working memory, inhibitory control, and goal maintenance and monitoring (Coull et al., 1996; Hilti et al., 2010). This may imply that these processes, associated with the broader cognitive domain of executive functioning, may differ across the subgroups.

Impairments in emotion recognition (ERT) were not observed in all FEP patients; cluster 4 (globally-normal) performed similar as healthy controls. Only cluster 3 (globally-poor) showed difficulties recognizing happiness, anger and disgust compared to controls. This is interesting, considering that cluster 3 is characterized by the poorest trajectory of PMF in the social domain. Although from the present study it is not clear whether these deficits in emotion recognition were already present before psychosis onset, this does raise the possibility that difficulties in premorbid social functioning may be associated with more widespread deficits in emotion recognition, including happiness, anger and disgust.

Some researchers have suggested that groups with different PMF profiles mainly differ on the severity of cognitive impairment, rather than the pattern of cognitive impairment (Quee et al., 2014). The present study broadly confirms this, but extends previous findings by demonstrating that aspects of emotion recognition may differentiate between subgroups of patients.

### 4.3 Symptom severity across clusters

The identification of four PMF profiles matches earlier observations in FEP (Larsen et al., 2004; Addington et al., 2005). In contrast to previous research (Addington and Addington, 2005), not all patients with a deteriorating course of PMF demonstrated more positive psychotic symptoms. Addington and Addington (2005) reported more severe negative symptoms and social functioning impairments in groups with a deteriorating and poor-deteriorating course of overall premorbid functioning. Our results indicate that more severe negative symptoms and social functioning impairments are present in groups with a poor or deteriorating PMF in both academic and social domains, but not in patients with poor or deteriorating academic PMF only. This supports the previously proposed subdivision of premorbid functioning into a social and academic component (Barajas et al., 2013; Chang et al., 2013; Ferraro et al., 2021).

Our findings suggest that premorbid functioning trajectories could be helpful in clinical practice for patient stratification. While those with more preserved trajectories may benefit from preventive measures meant to preserve functioning, identifying people with globally poor or declining premorbid profiles early in the course of illness may help prioritize intensive interventions, such as cognitive remediation and psychosocial support. In the end, this kind of stratification could enhance individualized treatment for first-episode psychosis by guiding resource allocation and service planning.

Two limitations of this study are missing data due to drop-out throughout the study period, as well as the retrospective assessment of premorbid functioning, which increases the chance of selective recall and memory bias in patients and family members. Prospective studies, following individuals over long time periods (starting from childhood) would be of great value, but are cost-intensive and even more sensitive to attrition bias. This study is also limited by the inability to control for the impact of antipsychotic medication on cognition, a factor that has been previously shown to account for a significant portion of the cognitive impairment in patients (Velthorst et al., 2021). In addition, although the standardized administration and non-verbal nature of the cognitive test battery fits well with the needs of this multi-center international study, the results may be less generalizable to verbal cognitive domains (e.g. verbal knowledge and memory). Since the strongest associations have been found between premorbid functioning and verbal learning and memory (Rund et al., 2004; Stefanatou et al., 2018; Addington et al., 2003; Jordan et al., 2018), future similar research with a greater focus on verbal measures of cognition is recommended. Another uncontrolled factor is the apparent practice effect observed across the groups. This is consistent with previous longitudinal studies using repeated cognitive testing (Watson et al., 2022). Although these improvements are unlikely to represent true cognitive recovery, they could mask subtle declines or group differences, particularly over the relatively brief 12-month follow-up interval. Future research using alternative testing paradigms and longer follow-up periods may help disentangle practice-related effects from actual cognitive change.

Taken together, our results suggest that four trajectories of premorbid functioning can be distinguished, which are related to the severity and trajectories of cognitive functioning after a first-episode psychosis. We specifically provided new insight into the possible link between premorbid functioning trajectories and the cognitive domain of emotion recognition. The clusters of premorbid functioning were mainly characterized by different *levels* of cognitive impairments, rather than a different *course*, at least in the short term. Premorbid functioning being recognized as an indicator of cognitive reserve, our findings add to the existing evidence that cognitive reserve could explain the heterogeneity in cognitive outcomes in FEP. Future similar studies, using longer follow-up periods, and comparing neurobiological measures between subgroups with different premorbid functioning profiles, are required to validate our findings and to better understand the origin of differences in premorbid functioning. This could eventually help in early identification of patients at risk of poorer outcomes, who may need different or more intensive treatment.

## Glossary

CGI-S: Clinical Global Impression - Severity
DSM: Diagnostic and Statistical Manual of Mental Disorders
DUP: Duration of untreated psychosis
ERT: Emotion Recognition Task
FEP: First-episode psychosis
GAF: Global Assessment of Functioning
HC: Healthy control
HDRS: Hamilton Depression Rating Scale
PAL: Paired Associate Learning Task
PAS: Premorbid Adjustment Scale
PANSS: Positive and Negative Syndrome Scale
PMF: Premorbid functioning
RVP: Rapid Visual Information Processing Task
SOFAS: Social and Occupational Functioning Assessment Scale
SSP: Spatial Span Task
WAIS: Wechsler Adult Intelligence Scale

## CRediT authorship contribution statement

**Margot I.E. Slot:** Writing – review & editing, Writing – original draft, Visualization, Validation, Methodology, Investigation, Formal analysis, Data curation, Conceptualization. **Hendrika H. van Hell:** Writing – review & editing, Supervision, Resources, Project administration, Investigation, Data curation, Conceptualization. **Inge Winter-van Rossum:** Writing – review & editing, Supervision, Resources, Project administration, Investigation, Conceptualization. **George Gifford:** Writing – review & editing, Resources, Data curation. **Paola Dazzan:** Writing – review & editing, Resources, Investigation. **Arija Maat:** Writing – review & editing, Resources, Investigation. **Lieuwe De Haan:** Writing – review & editing, Resources, Investigation. **Benedicto Crespo-Facorro:** Writing – review & editing, Resources, Investigation. **Birte Y. Glenthøj:** Writing – review & editing, Resources, Investigation. **Colm McDonald:** Writing – review & editing, Resources, Investigation. **Thérèse van Amelsvoort:** Writing – review & editing, Resources, Investigation. **Celso Arango:** Writing – review & editing, Resources, Investigation. **Irina Falkenberg:** Writing – review & editing, Resources, Investigation. **Barnaby Nelson:** Writing – review & editing, Resources, Investigation. **Silvana Galderisi:** Writing – review & editing, Investigation, Conceptualization. **Mark Weiser:** Writing – review & editing, Investigation, Conceptualization. **Gabriele Sachs:** Writing – review & editing, Resources, Investigation. **Anke Maatz:** Writing – review & editing, Resources, Investigation. **Jun Soo Kwon:** Writing – review & editing, Resources, Investigation. **Philip McGuire:** Writing – review & editing, Supervision, Resources, Project administration, Methodology, Investigation, Funding acquisition, Conceptualization. **René S. Kahn:** Writing – review & editing, Supervision, Resources, Project administration, Methodology, Investigation, Funding acquisition, Conceptualization.

## Funding sources

PSYSCAN was supported by the 10.13039/501100000780European Commission within its 7th Framework Programme (grant agreement no 603196).

## Declaration of competing interest

The authors declare the following financial interests/personal relationships which may be considered as potential competing interests: B.Y.G. has been the leader of a Lundbeck Foundation Centre of Excellence for Clinical Intervention and Neuropsychiatric Schizophrenia Research (CINS) (January 2009–December 2021), which was partially financed by an independent grant from the Lundbeck Foundation based on international review and partially financed by the Mental Health Services in the Capital Region of Denmark, the University of Copenhagen, and other foundations. All grants are the property of the Mental Health Services in the Capital Region of Denmark and administrated by them. She has no other conflicts to disclose. S.G. has been a consultant and/or advisor to or has received honoraria from Angelini, Boehringer-Ingelheim, Gedeon Richter-Recordati, Janssen, Lundbeck, Otsuka, ROVI and Bristol Myers Squibb. The other authors declare none. If there are other authors, they declare that they have no known competing financial interests or personal relationships that could have appeared to influence the work reported in this paper.

## Data Availability

The data used in this study are available from the corresponding authors upon reasonable request.

## Appendix A Supplementary data

Supplementary material Image 1
