# Supplementary material for: Premorbid functioning trajectories and the one-year course of cognitive performance in first-episode psychosis: a cluster analysis in PSYSCAN
Source: Schizophr Res Cogn. 2025 Sep 26;43:100391. doi: 10.1016/j.scog.2025.100391 (PMC12508902; doi:10.1016/j.scog.2025.100391)
Supplement: Supplementary file 1 — Supplementary material [file mmc1.docx]

# Supplements

Premorbid functioning trajectories and the one-year course of cognitive performance in first-episode psychosis: a longitudinal cluster analysis in PSYSCAN.

Authors:

Margot I.E. Slot ^1^, Hendrika H. van Hell ^1^, Inge Winter-van Rossum ^1,2,^^3^, George Gifford ^3^, Paola Dazzan ^4^, Arija Maat ^1^, Lieuwe De Haan ^5^, Benedicto Crespo-Facorro ^6^, Birte Y. Glenthøj ^7,8^, Colm McDonald ^9^, Thérèse van Amelsvoort ^10^, Celso Arango ^11^, Irina Falkenberg ^12^, Barnaby Nelson ^13,14^, Silvana Galderisi ^15^, Mark Weiser ^16,17^, Gabriele Sachs ^18^, Anke Maatz ^19^, Jun Soo Kwon ^20,21,22^, Philip McGuire ^3^, René S. Kahn ^1,2^.

1 Department of Psychiatry, UMC Utrecht Brain Center, University Medical Center Utrecht, Utrecht, The Netherlands

2 Department of Psychiatry, Icahn School of Medicine, Mount Sinai, New York, The United States of America

3 Department of Psychiatry, University of Oxford, Warneford Hospital, Oxford, United Kingdom

5 Amsterdam UMC, University of Amsterdam, Psychiatry, Department Early Psychosis, Amsterdam, Meibergdreef 9, The Netherlands

6 Hospital Universitario Virgen del Rocio, CIBERSAM, IBiS-CSIC. Department of Psychiatry, School of Medicine, University of Sevilla, Spain

7 Centre for Neuropsychiatric Schizophrenia Research (CNSR) & Centre for Clinical Intervention and Neuropsychiatric Schizophrenia Research (CINS), Mental Health Centre Glostrup, Glostrup, Denmark

8 University of Copenhagen, Faculty of Health and Medical Sciences, Department of Clinical Medicine, Copenhagen, Denmark

9 Centre for Neuroimaging & Cognitive Genomics (NICOG), NCBES Galway Neuroscience Centre, National University of Ireland Galway, H91 TK33, Galway, Ireland

10 Department of Psychiatry and Neuropsychology, Maastricht University , Maastricht, The Netherlands

11 Department of Child and Adolescent Psychiatry, Institute of Psychiatry and Mental Health, Hospital General Universitario Gregorio Marañón, IiSGM, CIBERSAM, ISCIII, School of Medicine, Universidad Complutense, Madrid, Spain

12 Department of Psychiatry, University of Marburg, Rudolf-Bultmann-Straße 8, D-35039, Marburg, Germany

13 Orygen, Melbourne, VIC, Australia

14 Centre for Youth Mental Health, University of Melbourne, Melbourne, VIC, Australia

15 Department of Mental and Physical Health and Preventive Medicine, University of Campania L. Vanvitelli, Naples, Italy

16 Zachai Department of Psychiatry, Sheba Medical Center, Tel Hashomer 52621, Israel

17 Tel Aviv University School of Medicine, Ramat Aviv, Israel

18 Department of Psychiatry and Psychotherapy, Medical University of Vienna, 1090 Vienna, Austria

19 Department of Adult Psychiatry and Psychotherapy, Psychiatric University Clinic Zurich and University of Zurich

20 Department of Psychiatry, Hanyang University Hospital, 222-1 Wangsimni-ro, Seongdong-gu, Seoul, Republic of Korea

21 Department of Neuropsychiatry, Seoul National University Hospital, 101 Daehakno, Jongno-gu, Seoul, Korea

22 Institute of Human Behavioral Medicine, SNU-MRC, 101 Daehakno, Jongno-gu, Seoul, Republic of Korea

## Supplementary Methods 1. Description cognitive test battery.

*Emotion recognition*

The Emotion Recognition Task (ERT) was used to measure the participant’s ability to identify the basic emotions sadness, happiness, fear, anger, disgust and surprise in facial expressions. Facial images derived from the facial features of real individuals were displayed on the screen, one at a time, each showing a specific emotion. Facial expressions were displayed with 15 levels of intensity per emotion (1 being closest to neutral and 15 being the full expression of the emotion). In two series of 90 trials, faces were displayed for 200 milliseconds, after which it was masked for 250 milliseconds. Then, six buttons were displayed and the participant had to select the emotion that most closely corresponds to the shown facial expression. The outcome measure was the total number of correct answers across trials and across all emotions.

*Associative learning*

The Paired Associate Learning Task (PAL) was used to assess visuospatial episodic memory and associative learning. Boxes are displayed on the screen and are opened in a randomized order. One or more of these boxes contain a pattern. After opening the boxes, the patterns are displayed in the center of the screen, one at a time. The participants are asked to select the box in which the pattern was originally located. The level of difficulty increases throughout the test by displaying either two, four, six or eight patterns. If an error is made, the boxes are opened in sequence again to remind the participant of the pattern locations. The outcome measure is the number of times the participant chose the incorrect box for a pattern on assessment problems, adjusted for the estimated number of errors he would have made on any attempts, recalls and problems he did not reach.

*Working memory*

The Spatial Span Task (SSP), a computerized version of the Corsi block test (Corsi, 1972), was used to assess visual working memory. White squares are displayed on the screen, some of which briefly change color in a variable sequence. The number of boxes in the sequence increases from two to nine, and the sequence and color are varied throughout the test. The participant must select the boxes which changed color in the same order that they were displayed by the computer (forward variant) or in the reverse order (backward variant). The outcome variable is the longest sequence of spatial items successfully recalled (‘forward spatial span length’ and ‘reverse spatial span length’).

*Sustained attention*

The Rapid Visual Information Processing Task (RVP) was used to assess sustained visual attention. Digits from 2 to 9 appear in a pseudo-random order in a white box shown in the middle of the screen. Participants are asked to detect target sequences of digits (e.g., 2-4-6). When the participant sees the target sequence, he must respond by touching the button in the center of the screen as quickly as possible. The main outcome variable of this task is A prime (A’), the signal detection measure of a participant’s sensitivity to the target sequence; i.e., it evaluates the ability of an individual to distinguish target sequences (strings of three numbers) from noise (non-target sequences). Scores range from 0-1 (higher scores indicating better performance).

## **Supplementary Table 1.** Eligibility criteria FEP and HC cohort PSYSCAN.

|  | *FEP cohort* | *HC cohort* |
| --- | --- | --- |
| **Inclusion criteria** | - Age 16–40 years - Capable of providing informed consent (or assent in case of a minor) - A first episode of psychosis as defined by a DSM-IV diagnosis of schizophrenia, schizoaffective disorder (depressive type) or schizophreniform disorder (Diagnostic and Statistical Manual of Mental Disorders, 4th ed., text rev.) (American Psychiatric Association, 2000) | - Age 16–40 years - Capable of providing informed consent (or assent in case of a minor) |
| **Exclusion criteria** | - A time interval between psychosis onset and study entry exceeding three years; onset of psychosis was defined as the initiation of treatment for psychosis (date of hospital admission or acceptance at healthcare service for psychosis) - Any previous neurosurgery or neurological disorder, including epilepsy - A history of head injury resulting in unconsciousness lasting at least 1 hour - Pregnancy - Any contraindications for MRI - Refusing to undergo blood draws and/or MRI sessions - Unable to fully comprehend the purpose of the study or to make a rational decision on whether or not to participate | - Any previous neurosurgery or neurological disorder, including epilepsy - A history of head injury resulting in unconsciousness lasting at least 1 hour - Pregnancy - Any contraindications for MRI - Refusing to undergo blood draws and/or MRI sessions - Unable to fully comprehend the purpose of the study or to make a rational decision on whether or not to participate - Lifetime history of a DSM Axis-I psychiatric disorder - At high risk of developing psychosis, based on inclusion in one of three groups as assessed by the CAARMS (version 2006; Yung et al., 2005) and meeting criteria for “basic symptoms” as assessed using the SPI-A (Schultze-Lutter et al., 2006) - First-degree relative with a lifetime history of affective or non-affective psychosis (defined by treatment or diagnosis) - Previous intake of antipsychotic medication - Current intake of psychoactive medication - IQ < 70 |

## **Supplementary Table 2.** Sample questions short PAS clinical interview.

|  | *Sample questions* |
| --- | --- |
| **Childhood (up to age 11)** | 1. Establish time period and any major life events at this stage: i.e. Which junior/infants school(s) did you go to at this time? Describe what you were like at this age. 2. In general, how did you get on at school; did you find it enjoyable and interesting? 3. How did you find the work? Were you at the top, bottom or middle of your class? If ‘streamed’ (according to ability) which groups were you in? 4. How did you get on with other students and teachers? 5. Were you a member of any teams, clubs or groups at this school? 6. Did you ever get into trouble at school? Did you play truant or refuse to go to school? 7. Did you have many friends? Were these close/best friends or casual friends? Were your friends about the same age as you? 8. How regularly did you see these friends? 9. Did you spend much time on your own at this age? 10. Were you a shy person at this age? Would you approach other children to talk to or play with or did you usually wait until others asked you to join in? 11. At this age did you spend much time day dreaming or ‘in your own world’? |
| **Early adolescence (ages 12-16)** | 1. Establish time period and any major life events during this period. (i.e. Which secondary school(s) did you go to at this time?) In what ways did you change as you got older? 2. In general, how did you get on at school; did you find it interesting and enjoyable? 3. How did you find the work? Were you at the top, bottom or middle of your class? If ‘streamed’ (according to ability) which groups were you in? 4. How did you get on with other students and teachers? 5. Were you a member of any teams, clubs or groups at this school? 6. Did you get into trouble at school? Did you play truant or refuse to go to school? 7. Did you have many friends? Were these close/best friends or casual friends? Were your friends about the same age as you? 8. How regularly did you see these friends? 9. Did you spend much time on your own at this age? 10. Were you a shy person at this age? Would you approach other people or did you wait until others asked you to join them? 11. At this age did you spend time daydreaming or ‘in your own world’? 12. Did you have male/female (use opposite gender to the client) friends at this age? 13. Did you ever go out on dates? Did you have a boyfriend(s) / girlfriend(s)? 14. Did you show physical signs of affection such as hugging or kissing? Did you have a sexual relationship with any of your girlfriends/boyfriends? 15. (If person did not date at this age) Were there boys/girls who you liked/were interested in/fancied? |

## Supplementary Table 3. Excluded cognitive assessments due to researcher observations.

|  |  |  | Distraction (n) | Technical issue  (n) | Refusal  (n) | Did not understand instructions  (n) | Deviation from instruction  (n) | Non-adherence  (n) | Other  (n) | Multiple reasons  (n) | Total N |
| --- | --- | --- | --- | --- | --- | --- | --- | --- | --- | --- | --- |
|  |  |  |  |  |  |  |  |  |  |  |  |
| **PAL** | Month 0 | FEP | 1 | 2 | 0 | 0 | 0 | 0 | 0 | 0 | 3 |
|  |  | HC | 0 | 0 | 0 | 0 | 0 | 0 | 0 | 0 | 0 |
|  | Month 6 | FEP | 2 | 1 | 1 | 0 | 0 | 0 | 0 | 0 | 4 |
|  |  | HC | 0 | 0 | 0 | 0 | 0 | 0 | 0 | 0 | 0 |
|  | Month 12 | FEP | 2 | 0 | 0 | 0 | 0 | 0 | 0 | 0 | 2 |
|  |  | HC | 0 | 0 | 0 | 0 | 0 | 0 | 0 | 0 | 0 |
| **RVP** | Month 0 | FEP | 13 | 4 | 2 | 13 | 1 | 2 | 4 | 3 | 42 |
|  |  | HC | 0 | 1 | 0 | 1 | 0 | 0 | 0 | 0 | 2 |
|  | Month 6 | FEP | 5 | 1 | 3 | 1 | 1 | 3 | 0 | 3 | 17 |
|  |  | HC | 0 | 0 | 0 | 0 | 0 | 0 | 1 | 0 | 1 |
|  | Month 12 | FEP | 4 | 0 | 0 | 1 | 0 | 1 | 0 | 0 | 6 |
|  |  | HC | 0 | 2 | 0 | 0 | 0 | 0 | 0 | 0 | 2 |
| **SSPF** | Month 0 | FEP | 1 | 2 | 0 | 0 | 0 | 2 | 3 | 1 | 9 |
|  |  | HC | 0 | 0 | 0 | 0 | 0 | 0 | 1 | 0 | 1 |
|  | Month 6 | FEP | 3 | 1 | 0 | 0 | 0 | 3 | 0 | 0 | 7 |
|  |  | HC | 0 | 0 | 0 | 0 | 0 | 0 | 0 | 0 | 0 |
|  | Month 12 | FEP | 2 | 0 | 0 | 0 | 0 | 1 | 0 | 0 | 3 |
|  |  | HC | 0 | 0 | 0 | 0 | 0 | 0 | 0 | 0 | 0 |
| **SSPR** | Month 0 | FEP | 2 | 1 | 0 | 0 | 0 | 2 | 3 | 0 | 8 |
|  |  | HC | 0 | 0 | 0 | 0 | 0 | 0 | 1 | 0 | 1 |
|  | Month 6 | FEP | 1 | 2 | 0 | 0 | 1 | 3 | 0 | 0 | 7 |
|  |  | HC | 0 | 0 | 0 | 0 | 0 | 0 | 0 | 0 | 0 |
|  | Month 12 | FEP | 3 | 1 | 0 | 0 | 0 | 1 | 0 | 0 | 5 |
|  |  | HC | 0 | 0 | 0 | 0 | 0 | 0 | 0 | 0 | 0 |
| **ERT** | Month 0 | FEP | 2 | 1 | 0 | 0 | 0 | 2 | 1 | 0 | 6 |
|  |  | HC | 0 | 0 | 0 | 0 | 0 | 0 | 0 | 0 | 0 |
|  | Month 6 | FEP | 2 | 0 | 0 | 0 | 0 | 3 | 1 | 0 | 6 |
|  |  | HC | 0 | 0 | 0 | 0 | 0 | 0 | 0 | 0 | 0 |
|  | Month 12 | FEP | 1 | 1 | 0 | 0 | 0 | 1 | 0 | 0 | 3 |
|  |  | HC | 0 | 0 | 0 | 0 | 0 | 0 | 0 | 0 | 0 |

*Note.* This table refers to subjects who completed at least one cognitive subtest and who had not yet been excluded based on age <18 years, a run time >45 minutes or the COVID-19 pandemic. PAL = Paired Associate Learning Task. RVP = Rapid Visual Information Processing Task. SSPF = Spatial Span Task – Forward. SSPR = Spatial Span Task – Reverse. ERT = Emotion Recognition Task.

## Supplementary Table 4. Sociodemographic and clinical characteristics of the PSYSCAN FEP and HC cohort.

|  | FEP cohort | HC cohort | Test | *p*-value |
| --- | --- | --- | --- | --- |
|  | *n = 302* | *n = 136* | statistic (df) |  |
| Sex |  |  | χ^2^(1)= 2.25 | 0.133 |
| Female | 99 (32.8%) | 55 (40.7%) |  |  |
| Male | 203 (67.2%) | 81 (59.6%) |  |  |
| Age (years) | 25.3 (5.7) | 23.8 (4.3) | *t*(331.42) = 3.12 | 0.002 |
| Years of education * | 14.2 (3.1) | 16.0 (3.0) | *t*(434) = -5.70 | < 0.001 |
| Educational level ** |  |  |  | . |
| Less than high school | 15 (5.0%) | 0 (0.0%) |  |  |
| High school | 117 (38.7%) | 14 (10.5%) |  |  |
| Professional training | 78 (25.8%) | 24 (18.0%) |  |  |
| University | 83 (27.5%) | 76 (57.1%) |  |  |
| Post-graduate university | 9 (3.0%) | 19 (14.3%) |  |  |
| Employment |  |  | χ^2^(1) = 64.13 | < 0.001 |
| Employed or student | 148 (49.0%) | 104 (92.9%) |  |  |
| Unemployed | 154 (51.0%) | 8 (7.1%) |  |  |
| Time since treatment initiation for  psychosis (months), *Mdn (IQR)*^†^ | 9.4 (9.3) |  |  |  |
| DUP (months), *Mdn (IQR)*^‡^ | 5.9 (16.6) |  |  |  |
| PANSS total | 55.2 (16.8) | 31.7 (2.2) | *t*(320.90) = 23.68 | < 0.001 |
| PANSS positive | 13.1 (5.6) | 7.1 (0.4) | *t*(305.41) = 18.30 | < 0.001 |
| PANSS negative | 14.3 (6.3) | 7.3 (0.8) | *t*(317.37) = 18.99 | < 0.001 |
| PANSS general | 27.8 (8.3) | 17.3 (1.8) | *t*(357.17) = 20.71 | < 0.001 |
| SOFAS score | 54.4 (17.9) | 85.2 (6.5) | *t*(418.68) = -26.08 | < 0.001 |
| WAIS total IQ | 90.8 (18.6) | 112.2 (16.0) | *t*(421) = -11.48 | < 0.001 |
| WAIS arithmetic | 8.4 (3.7) | 11.3 (3.8) | *t*(422) = -6.47 | < 0.001 |
| WAIS symbol substitution | 7.6 (3.0) | 11.2 (3.1) | *t*(422) = -11.18 | < 0.001 |
| WAIS information | 10.0 (3.9) | 12.0 (3.3) | *t*(293.31) = -5.42 | < 0.001 |
| WAIS block design | 8.7 (3.9) | 12.3 (3.0) | *t*(328.72) = -10.38 | < 0.001 |

*Note.* Data are n (%) or mean (SD), unless otherwise indicated. P-value of t-test (continuous variable normal distributed), Mann-Whitney test (continuous variable non-normal distributed) or Chi-Square test (categorical variable). * Years of education = years in school and college/university (not including kindergarten/nursery). ** Each category reflects individuals who finished as well as individuals who did not (yet) finish this level of education. ^†^ Treatment initiation is defined as the date of first acceptance at healthcare service for psychosis (this could be either inpatient or outpatient, depending on the setting to which the individual first presented for psychosis). ^‡^ Duration of untreated psychosis (DUP) is defined as the time interval between first onset of frank psychotic symptoms and the date of first acceptance at healthcare service for psychosis. FEP = First Episode Psychosis in the context of a Schizophrenia-Spectrum Disorder. PANSS = Positive and Negative Syndrome Scale (score range: positive (7-49), negative (7-49), general (16-112), total (30-210); higher scores indicate more severe psychotic symptoms). SOFAS = Social and Occupational Functioning Assessment Scale (1-100, higher scores indicate a higher level of functioning). WAIS-III SF = Wechsler Adult Intelligence Scale, Third Edition, Short Form.

## Supplementary Table 5. Linear mixed model results of longitudinal changes in cognitive performance in FEP participants and healthy controls (HC).

| **Outcome variable** | **Primary analyses** | | |  |  | | **Pairwise comparisons** | | |
| --- | --- | --- | --- | --- | --- | --- | --- | --- | --- |
|  | *F*-statistic | Df | *p*-value | Partial *η²* | |  | *t*-statistic | Df | *p*-value |
| RVP | Time point * group: 3.77  Educational years: 25.49 | 2, 491.1  1, 388.1 | .0237  < .0001 | 0.01  0.06 | |  | Month 0 (FEP) < Month 6 (FEP) -4.5  Month 0 (FEP) < Month 12 (FEP) -6.8  Month 0 (HC) < Month 6 (HC) -6.0  Month 0 (HC) < Month 12 (HC) -7.9  FEP < HC (Month 0) -7.1  FEP < HC (Month 6) -8.3  FEP < HC (Month 12) -8.6 | 503  523  493  499  563  675  686 | .0001  < .0001  < .0001  < .0001  < .0001  < .0001  < .0001 |
| PAL | Time point: 7.54  Group: 75.46  Educational years: 4.88 | 2, 536.4  1, 392.6  1, 394.6 | .0006  < .0001  .0277 | 0.03  0.17  0.01 | |  | Month 0 < Month 12 -3.8 | 560 | .0004 |
| SSP forward | Time point: 1.38  Group: 77.00  Educational years: 8.17 | 2, 551.4  1, 375.0  1, 377.0 | .2519  < .0001  .0045 | 0.0049  0.17  0.02 | |  |  |  |  |
| SSP backward | Time point: 65.98  Group: 4.62  Educational years: 9.55 | 2, 543.5  1, 384.0  1, 385.7 | .0102  < .0001  .0021 | 0.02  0.15  0.02 | |  | Month 0 < Month 12 -3.0 | 565 | .0072 |
| ERT | Time point: 1.95  Group: 13.24  Educational years: 20.47 | 2, 568.1  1, 395.3  1, 396.6 | .1437  .0003  < .0001 | 0.0060  0.04  0.05 | |  |  |  |  |

*Note.* Analyses are corrected for years of education. Pairwise contrasts were corrected for multiple comparisons using the Tukey method. Only significant

pairwise comparisons are shown. RVP = Rapid Visual Information Processing Task. PAL = Paired Associate Learning Task. SSP = Spatial Span Task. ERT = Emotion
Recognition Task.

## Supplementary Table 6. Results of linear mixed model analyses on the total number of hits on the different emotions of the ERT (raw scores), with PAS cluster membership as between-subject factor (group), time point as a repeated measures within-subject factor (time point) and sex as covariate.

| **Outcome variable** | **Primary analyses** | | |  | **Pairwise comparisons** | | |
| --- | --- | --- | --- | --- | --- | --- | --- |
|  | *F*-statistic | Df | *p*-value |  | *t*-statistic | Df | *p*-value |
| ERT - Happiness | Time point: 4.70  Group: 2.96  Sex: 0.00 | 2, 590.5  4, 376.4  1, 375.0 | .0095  .0198  .9742 |  | Month 0 > Month 12 2.8  Month 6 > Month 12 2.6  Cluster 3 < HC cohort -2.8 | 620  585  380 | .0161  .0256  .0374 |
| ERT - Sadness | Time point: 0.77  Group: 8.40  Sex: 0.91 | 2, 603.1  4, 381.0  1, 379.4 | .4650  <.0001    .3414 |  | Cluster 1 < HC cohort -5.2  Cluster 2 < HC cohort -3.8  Cluster 3 < HC cohort -3.2  Cluster 1 < Cluster 4 -2.9 | 405  409  377  400 | <.0001  .0015  .0126  .0322 |
| ERT - Anger | Time point: 2.52  Group: 3.51  Sex: 2.84 | 2, 596.1  4, 393.3  1, 392.1 | .0812  .0078  .0926 |  | Cluster 3 < HC cohort -3.4  Cluster 1 > Cluster 3 2.7  Cluster 2 > Cluster 3 3.2  Cluster 4 > Cluster 3 2.8 | 384  392  395  386 | .0090  .0490  .0138  .0416 |
| ERT - Surprise | Time point: 0.55  Group: 4.93  Sex: 1.20 | 2, 602.8  4, 369.3  1, 367.4 | .5761  .0007  .2731 |  | Cluster 1 < HC cohort -3.3  Cluster 2 < HC cohort -3.6  Cluster 3 < HC cohort -2.9 | 403  407  372 | .0103  .0032  .0278 |
| ERT - Fear | Time point: 0.36  Group: 1.38  Sex: 0.26 | 2, 610.8  4, 395.9  1, 394.4 | .7006  .2407  .6077 |  |  |  |  |
| ERT - Disgust | Time point: 2.48  Group: 5.48  Sex: 3.90 | 2, 599.3  4, 395.5  1, 394.2 | .0844  .0003  .0491 |  | Cluster 3 < HC cohort -4.6 | 384 | .0001 |

*Note.* Pairwise contrasts were corrected for multiple comparisons using the Tukey method. Only significant pairwise comparisons and trends are shown,
with p-values for trends indicated in brackets (). Results reflect exploratory analyses of raw scores (uncorrected for sex and age) due to the unavailability of
normative scores for the analysis of the different types of emotion on the ERT.

## **Supplementary Figure 1.** The course of cognitive performance over a one-year period in FEP participants and healthy controls (HC). Results of repeated measures mixed model analyses, corrected for years of education. Whiskers represent 95% confidence intervals.

Supplementary Figure 2. Results of linear mixed model analyses on the total number of hits on the different emotions of the ERT (raw scores), with PAS cluster membership as between-subject factor (group), time point as a repeated measures within-subject factor (time point) and sex as covariate. Whiskers represent 95% confidence intervals.

## Names PSYSCAN Consortium members

*London:*

Philip McGuire ^3, 45^

Stefania Tognin ^45^

Paolo Fusar-Poli ^45, 46, 47, 48^

Matthew J. Kempton ^45^

Alexis E. Cullen ^45, 49^

Gemma Modinos ^6^

Kate Merritt ^45, 50^

Andrea Mechelli ^45^

Paola Dazzan ^6^

George Gifford ^45^

Natalia Petros ^45^

Mathilde Antoniades ^45^

Andrea De Micheli ^45^

Sandra Vieira ^45, 51, 52^

Tom Spencer ^45, 47^

Zhaoying Yu ^45^

Dominic Oliver ^3, 45^

Fiona Coutts ^45^

Emily Hird ^53, 45^

Helen Baldwin ^54, 45^

*Utrecht:*

Rene Kahn ^1, 2^

Arija Maat ^1^

Erika van Hell ^1^

Inge Winter ^1, 2, 3^

Margot I.E. Slot ^1^

*Amsterdam:*

Lieuwe de Haan ^7^

Frederike Schirmbeck ^7^

*Cantabria:*

Benedicto Crespo-Facorro ^44^

Diana Tordesillas-Gutierrez ^8, 9^

Esther Setien-Suero ^8, 9^

Rosa Ayesa-Arriola ^8, 9^

Paula Suarez-Pinilla ^8, 9^

Victor Ortiz Garcia-de la foz ^8, 9^

*Copenhagen:*

Birte Glenthøj ^10, 11^

Mikkel Erlang Sørensen ^10^

Bjørn H. Ebdrup ^10, 11^

Jayachandra Mitta Raghava ^10, 11^

Egill Rostrup ^10, 25^

*Edinburgh:*

Stephen M. Lawrie ^12^

*Galway:*

Colm McDonald ^13^

Brian Hallahan ^13^

Dara M. Cannon ^13^

James McLoughlin ^13^

Martha Finnegan ^13^

*Heidelberg:*

Oliver Gruber ^14^

Anja Richter ^14^

Bernd Krämer ^14^

*Maastricht:*

Thérèse van Amelsvoort ^15^

Bea Campforts ^15^

Machteld Marcelis ^15, 16^

Claudia Vingerhoets ^15^

*Madrid:*

Celso Arango ^17^

Covadonga M. Díaz-Caneja ^17^

Miriam Ayora ^17^

Joost Janssen ^17^

Mara Parellada ^17^

Jessica Merchán-Naranjo ^17^

Roberto Rodríguez-Jiménez ^26^

Marina Díaz-Marsá ^27^

*Marburg:*

Tilo Kircher ^18^

Irina Falkenberg ^18^

Florian Bitsch ^18^

Jens Sommer ^18^

*Melbourne:*

Barnaby Nelson ^4, 5^

Patrick McGorry ^4, 5^

Paul Amminger ^4, 5^

Christos Pantelis ^4, 5^

Meredith McHugh ^4, 5^

Jessica Spark ^4, 5^

*Naples:*

Silvana Galderisi ^19^

Armida Mucci ^19^

Paola Bucci ^19^

Giuseppe Piegari ^19^

Daria Pietrafesa ^19^

Alessia Nicita ^19^

Sara Patriarca ^19^

*Tel Hashomer:*

Mark Weiser ^20, 21^

Linda Levi ^20, 21^

Yoav Domany ^20, 21^

*Vienna:*

Gabriele Sachs ^22^

Matthäus Willeit ^22^

Marcena Lenczowska ^22^

Ulrich Sauerzopf ^22^

Ana Weidenauer ^22^

Julia Furtner ^28^

Daniela Prayer ^22^

*Zurich:*

Anke Maatz ^29^

Matthias Kirschner ^23, 24^

Achim Burrer ^24^

Philipp Stämpfli ^24^

Naemi Huber ^24^

Stefan Kaiser ^23^

Wolfram Kawohl ^30^

*Sao Paulo:*

Rodrigo Bressan ^31^

André Zugman ^31^

Ary Gadelha ^31^

Graccielle Rodrigues da Cunha ^31^

*Seoul:*

Jun Soo Kwon ^32, 33^

Kang Ik Kevin Cho ^34, 35^

Taeyoung Lee ^33, 36^

Minah Kim ^37, 38^

Sun Young Moon ^37, 39^

Silvia Kyungjin Lho ^37, 40^

*Toronto/Montreal:*

Romina Mizrahi ^41^

Michael Kiang ^42, 43^

## Affiliations PSYSCAN Consortium members

1. Department of Psychiatry, UMC Utrecht Brain Center, University Medical Center Utrecht, Utrecht, The Netherlands
2. Department of Psychiatry, Icahn School of Medicine, Mount Sinai, New York, The United States of America
3. Department of Psychiatry, Division of Medical Sciences, University of Oxford, Warneford Hospital, OX3 7JX
4. Orygen, Melbourne, VIC, Australia
5. Centre for Youth Mental Health, University of Melbourne, Melbourne, VIC, Australia
6. Department of Psychological Medicine, Institute of Psychiatry, Psychology & Neuroscience, King's College London, De Crespigny Park, Denmark 458 Hill, London, United Kingdom SE5 8AF
7. Amsterdam UMC, University of Amsterdam, Psychiatry, Department Early Psychosis, Meibergdreef 9, Amsterdam, The Netherlands
8. Department of Psychiatry, Marqués de Valdecilla University Hospital, IDIVAL. School of Medicine, University of Cantabria, Santander, Spain
9. CIBERSAM, Centro Investigación Biomédica en Red Salud Mental, Spain
10. Centre for Neuropsychiatric Schizophrenia Research (CNSR) & Centre for Clinical Intervention and Neuropsychiatric Schizophrenia Research (CINS), Mental Health Centre Glostrup, Glostrup, Denmark
11. University of Copenhagen, Faculty of Health and Medical Sciences, Department of Clinical Medicine, Copenhagen, Denmark
12. Division of Psychiatry, University of Edinburgh, Royal Edinburgh Hospital, Edinburgh EH10 5HF, UK
13. Centre for Neuroimaging & Cognitive Genomics (NICOG), NCBES Galway Neuroscience Centre, National University of Ireland Galway, H91 TK33 Galway, Ireland
14. Section for Experimental Psychopathology and Neuroimaging, Department of General Psychiatry, Heidelberg University, Heidelberg, Germany
15. Department of Psychiatry and Neuropsychology, Maastricht University, Maastricht, The Netherlands
16. GGZE Mental Health Care, Eindhoven, the Netherlands
17. Department of Child and Adolescent Psychiatry, Institute of Psychiatry and Mental Health, Hospital General Universitario Gregorio Marañón, IiSGM, CIBERSAM, ISCIII, School of Medicine, Universidad Complutense, Madrid, Spain
18. Department of Psychiatry, University of Marburg, Rudolf-Bultmann-Straße 8, D-35039, Marburg, Germany
19. Department of Mental and Physical Health and Preventive Medicine, University of Campania Luigi Vanvitelli, Largo Madonna delle Grazie, 80138, Naples, Italy
20. Zachai Department of Psychiatry, Sheba Medical Center, Tel Hashomer 52621, Israel
21. Tel Aviv University School of Medicine, Ramat Aviv, Israel
22. Department of Psychiatry and Psychotherapy, 1090 Vienna, Austria
23. Division of Adult Psychiatry, Department of Psychiatry, University Hospitals of Geneva, Switzerland
24. Department of Psychiatry, Psychotherapy and Psychosomatics, Psychiatric Hospital, University of Zurich, Switzerland
25. Functional Imaging Unit (FIUNIT), Rigshospitalet Glostrup, University of Copenhagen, Glostrup, Denmark
26. Department of Psychiatry, Instituto de Investigación Sanitaria Hospital 12 de Octubre (imas 12), CIBERSAM, ISCIII, School of Medicine, Universidad Complutense, Madrid, Spain
27. Department of Psychiatry, Instituto de Investigación Hospital Clínico San Carlos (IdISSC), CIBERSAM, ISCIII, School of Medicine, Universidad Complutense, Madrid, Spain
28. Medical University of Vienna, Department of Biomedical Imaging and Image-guided Therapy Währingergürtel 18-20, 1090 Vienna
29. Department of Adult Psychiatry and Psychotherapy, Psychiatric University Clinic Zurich and University of Zurich
30. Department for Psychiatry and Psychotherapy, Psychiatric Services Aargau, Brugg, Switzerland
31. Department of Psychiatry, Interdisciplinary Lab for Clinical Neurosciences (LiNC), Universidade Federal de Sao Paulo (UNIFESP), Sao Paulo, Brazil
32. Department of Psychiatry, Hanyang University Hospital, 222-1 Wangsimni-ro, Seongdong-gu, Seoul, Republic of Korea
33. Institute of Human Behavioral Medicine, SNU-MRC, 101 Daehakno, Jongno-gu, Seoul, Republic of Korea
34. Department of Brain and Cognitive Sciences, Seoul National University College of Natural Sciences, Gwanakro1, Gwanak-gu, Seoul, Republic of Korea
35. Department of Psychiatry, Brigham and Women’s Hospital, Harvard Medical School, Boston, MA, USA
36. Department of Psychiatry, Kyungpook National University School of Medicine, Daegu, Republic of Korea
37. Department of Psychiatry, Seoul National University College of Medicine, 101 Daehakno, Jongno-gu, Seoul, Republic of Korea
38. Department of Neuropsychiatry, Seoul National University Hospital, 101 Daehakno, Jongno-gu, Seoul, Korea
39. Department of Public Health Medical Services, Seoul National University Bundang Hospital, Seongnam, Republic of Korea
40. Department of Psychiatry, Seoul Metropolitan Government-Seoul National University Boramae Medical Center, Seoul, Republic of Korea
41. Department of Psychiatry, McGill University, Montreal, Canada
42. Department of Psychiatry, University of Toronto, 250 College St 8th Floor, Toronto, Ontario, Canada M5T 1R8
43. Centre for Addiction and Mental Health, 250 College Street, Toronto, Ontario, Canada M5T 1R8
44. Hospital Universitario Virgen del Rocio, CIBERSAM, IBiS-CSIC. Department of Psychiatry, School of Medicine, University of Sevilla, Spain
45. Department of Psychosis Studies, Institute of Psychiatry, Psychology & Neuroscience, King's College London, De Crespigny Park, Denmark 458 Hill, London, United Kingdom SE5 8AF
46. Department of Brain and Behavioral Sciences, University of Pavia, Italy
47. Outreach and Support in South-London (OASIS) service, South London and Maudlsey (SLaM) NHS Foundation Trust, United Kingdom
48. Department of Psychiatry and Psychotherapy, Ludwig-Maximilian-University Munich, Germany
49. Division of Insurance Medicine, Department of Clinical Neuroscience, Karolinska Institutet, Sweden
50. MRC Centre for Neurodevelopmental Disorders, King's College London, United Kingdom
51. Department of Radiology, Lausanne University Hospital and University of Lausanne (CHUV-UNIL), Lausanne, Switzerland
52. Center for Research in Neuropsychology and Cognitive Behavioral Intervention, Faculty of Psychology and Educational Sciences, University of Coimbra, Coimbra, Portugal
53. Institute of Cognitive Neuroscience, Alexandra House, 17 Queen Square, London WC1N 3AZ
54. Health Service and Population Research (HSPR), Institute of Psychiatry, Psychology & Neuroscience, King's College London, De Crespigny Park, Denmark 458 Hill, London, United Kingdom SE5 8AF
